# Delineating Neuroanatomical Structures for the Measurement of Temporal Horn Dilatation

**DOI:** 10.21315/mjms-01-2025-077

**Published:** 2025-02-28

**Authors:** Devalagan Muthalagan, Kia Hooi Tan, Nur Asma Sapiai, Zul Izhar Mohd Ismail, Siti Fatimah Mukhtar, Wei Siong Ng, Siew Chung Mah

**Affiliations:** 1 Department of Neurosciences, School of Medical Sciences, Universiti Sains Malaysia, Health Campus, Kelantan, Malaysia; 2 Department of Radiology, School of Medical Sciences, Universiti Sains Malaysia, Health Campus, Kelantan, Malaysia; 3 Department of Anatomy, School of Medical Sciences, Universiti Sains Malaysia, Health Campus, Kelantan, Malaysia; 4 Clinical Neuropsychology Doctorate Trainees USM-UPSI Program, Department of Neurosciences, Hospital Pakar Universiti Sains Malaysia, Kelantan, Malaysia; 5 Brain and Behaviour Cluster, School of Medical Sciences, Universiti Sains Malaysia, Health Campus, Kelantan, Malaysia

Dear Editor,

We recently read with great interest the article by Zakaria et al. (1) entitled “The Key Aspects of Neonatal and Infant Neurological Examination: The Ballard Score, the Infant’s Head with Hydrocephalus and Assessment in a Clinical Setting”, in which the authors demonstrated clinical neonatal and neurological assessments in relation to hydrocephalus. We would like to further elaborate on the neuroimaging aspect of hydrocephalus, particularly highlighting that the temporal horn is typically the first ventricle to dilate in this condition (2). This anatomical feature makes its accurate measurement critical in both diagnosis and monitoring.

Temporal horn dilatation can be measured using at least two methods based on available literature, which we will describe here. We would like to further locate the neuroanatomical structures involved in deriving the points for the measurement used by these two distinct methods. It is important to note that both methods agree with the cut-off value of more than 2 mm in width to suggest dilatation of the temporal horns. The first method as described by Frisoni et al (3) and seen in Figure 1, involves measuring the radial width of the temporal horn (rWTH), which is the maximal transverse diameter of the horn, best visualised in an axial plane of the brain magnetic resonance imaging (MRI).

Based on the method described by Frisoni et al. (3), we can delineate the neuroanatomical structures on the axial views of computed tomography (CT) and MRI as such:

- Plane: Anterior Commissure-Posterior Commissure (AC-PC) line
- Anterior border: The anterior point corresponding to the anterior part of the collateral eminence (ce) which represents the most lateral aspect of the temporal horn (Figure 2, yellow star)
- Posterior border: The body of hippocampus (cornu Ammonis) which defines the medial boundary of the temporal horn (Figure 2, red arrow)[Fig f3-17mjms3201_le]

The second method in Figure 5 below, as described by Greenberg (5), is performed by drawing a tangential line passing through an anatomical point on the temporal horn on the axial plane, at the level of the petrous apex and subsequently measuring the largest distance between the two parallel lines.

Figure 4 showing ten contiguous 2-mm-thick CT axial slices (labelled A – J) of the temporal horn of lateral ventricles of a 3 years old child with grade 4 medulloblastoma that was causing obstructive hydrocephalus. By applying Frisoni’s method, and drawing tangential parallel lines across the anterior tip with the largest width (blue lines in J), neuroanatomically, this corresponds to the collateral eminence and the body of the hippocampus at the anterior and posterior points respectively. As such, the radial width of the temporal horn in this patient as depicted in J was 13.5 mm, which is well above the cut-off value of 2 mm – which would thus suggest temporal horn dilatation.

The anatomical point of these lines would correlate with the following landmarks:

- Level of image: Axial slice at level of temporal horns of the lateral ventricles, best seen at the level of petrous apex (5)
- Anterior Border: The amygdalohippocampal transition area (AHi), which delineates the hippocampus from the amygdala, marking the anterior extent of the temporal horn. (Figure 6, yellow arrow)[Fig f7-17mjms3201_le]
- Posterior Border: A line drawn parallel to the tangential line, at the posterior end

In measuring the temporal horn for dilatation using Greenberg’s method, the following steps are employed:

- The petrous apex (7) is first identified using the bone window on the coronal view, which serves as the anatomical landmark, as seen in Figure 8A
- Switch to the axial view in the brain window to visualise for dilatation of temporal horn as in Figure 8B
- A line is drawn across the petrous apex in the axial view. The anterior border of this line would correlate with the amygdalohippocampal transition area (AHi)
- Draw a second line that is parallel to the first line, encompassing the most lateral aspect of the temporal horn
- Measure the distance between the two lines to assess the temporal horn dilatation; in this case, the distance measures 9.6 mm (which exceeds 2 mm) and would support the definition of temporal horn dilatation as per Greenberg

These anatomical landmarks depicted above as described by Duvernoy et al, Schaltenbrand and Wahren (4, 6), can provide a reliable framework for temporal horn measurements. The reproducibility and reliability of these methods are crucial in both clinical and research settings, particularly for the accurate diagnosis and monitoring of hydrocephalus.

Accurate measurement of the temporal horn using the aforementioned methods provide critical insight into the progression of hydrocephalus and its impact on surrounding neuroanatomy. Hydrocephalus has impacted the surrounding neuroanatomy with the structural changes; specifically as the dilatation of the temporal horn lies in close proximity to critical structures involved in memory, emotional processing, and executive functions which includes the hippocampus and amygdala. The impact and changes could be associated with cognitive functions and clinical neuropsychological assessment outcomes of the individuals especially in those with congenital hydrocephalus and young age of having hydrocephalus. Research has shown that hydrocephalus can lead to deficits in attention, memory, processing speed, and executive function of the individuals (8). These deficits may be linked to the associated structural changes in the brain secondary to hydrocephalus.

As such, the early detection of temporal horn dilatation through neuroimaging with structural data is very important for understanding the physical changes in the brain and also for anticipating potential cognitive challenges. These data can help clinicians to highlight and monitor the neurodevelopmental changes over time and guide or breakthrough interventions aimed at improving cognitive function, such as neurocognitive rehabilitation, emotional and educational support. We believe these additional insights into the neuroimaging of the temporal horn, its dilatation, and measurements can contribute to the growing understanding of its diagnostic relevance in hydrocephalus. While the outlined methods provide a robust theoretical framework for measuring temporal horn dilatation, variations in imaging quality, patient anatomy, and observer’s interpretation may affect reproducibility. Future studies should aim to validate these methodologies in diverse clinical and demographic settings.

## Figures and Tables

**Figure 1:**
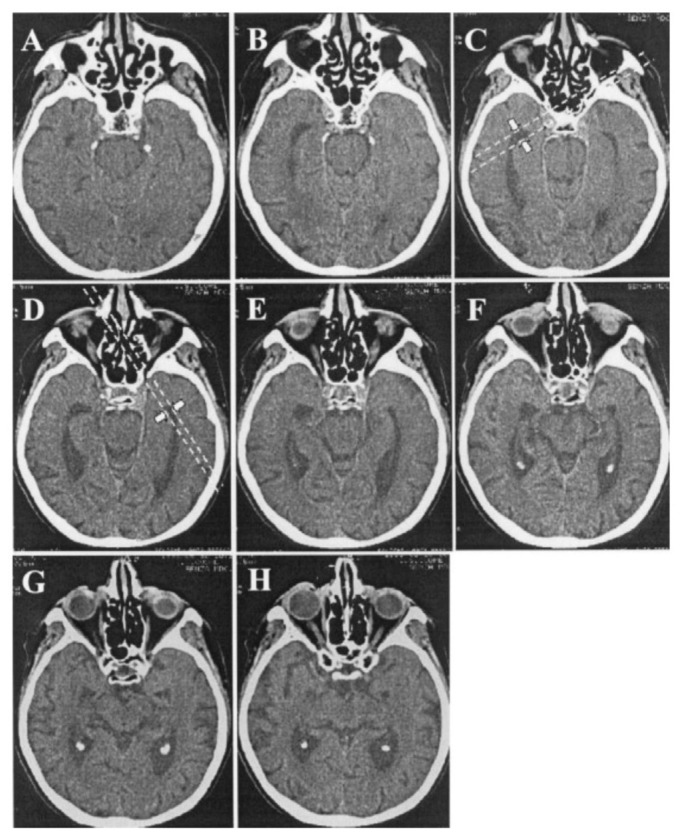
The measurement taken for transverse width of temporal horn Note: Adapted from Frisoni et al. (3); Parallel lines are drawn tangentially to the tip of the temporal horn where the width is maximum (arrows), at the level whereby the right and left temporal horns can be appreciated in their full length

**Figure 2:**
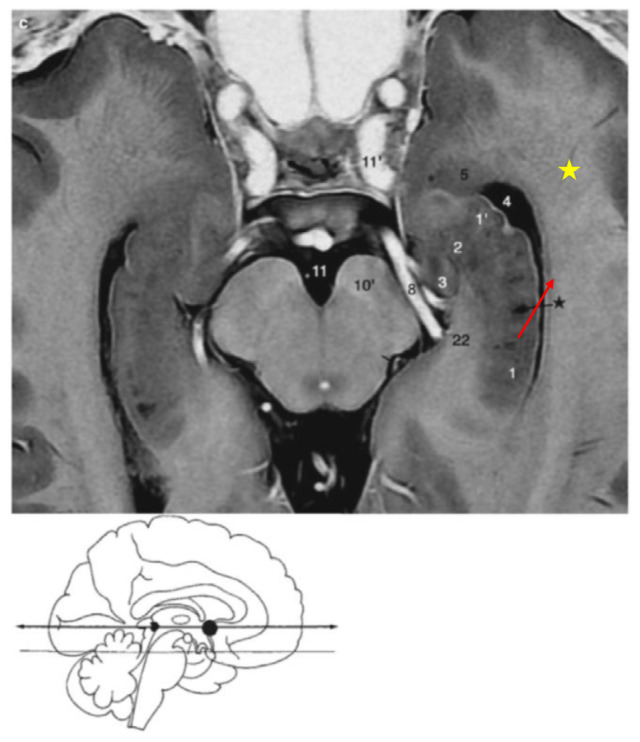
Head of hippocampus, uncal apex, temporal (inferior) horn of the lateral ventricle, amygdala, body of hippocampus, cornu Ammonis and collateral eminence (ce) Note: Adapted from Duvernoy et al. (Figure 7.26, pp. 212) (4); 1, 2 = head of hippocampus; 3 = uncal apex; 4 = temporal (inferior) horn of the lateral ventricle; 5 = amygdala; black star = body of hippocampus; red arrow = cornu Ammonis; yellow star = collateral eminence (ce)

**Figure 3 f3-17mjms3201_le:**
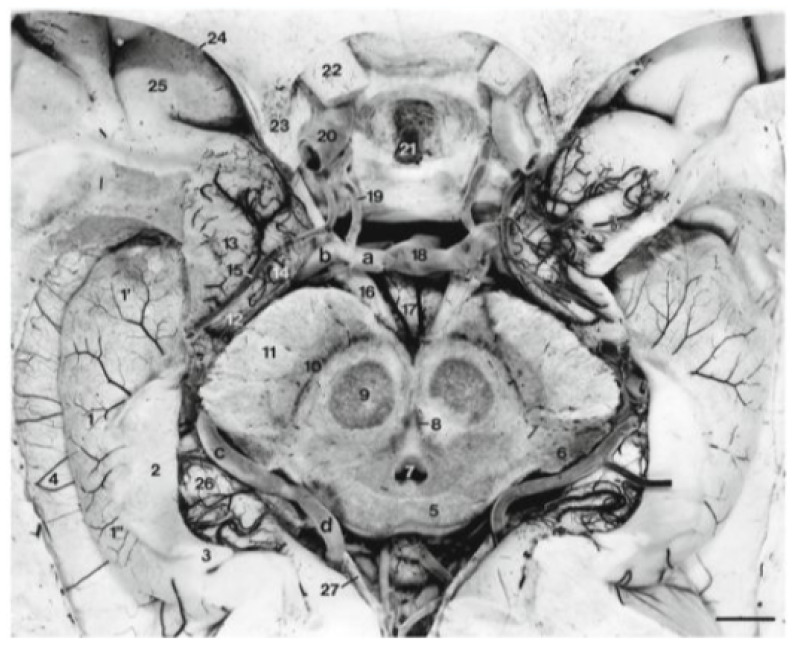
The collateral eminence, 4 bordering the lateral aspect of the hippocampus at the level of the upper mesencephalon, which constitutes the floor of the temporal horn. Note: Adapted from Duvernoy et al. (Figure 4.20, pp 63) (4)

**Figure 4:**
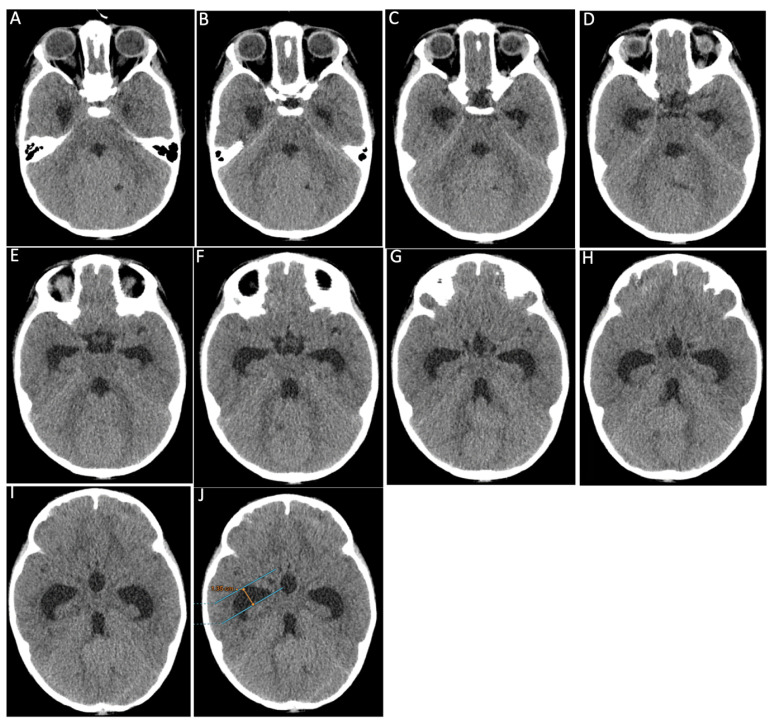
Ten contiguous 2-mm-thick CT axial slices

**Figure 5:**
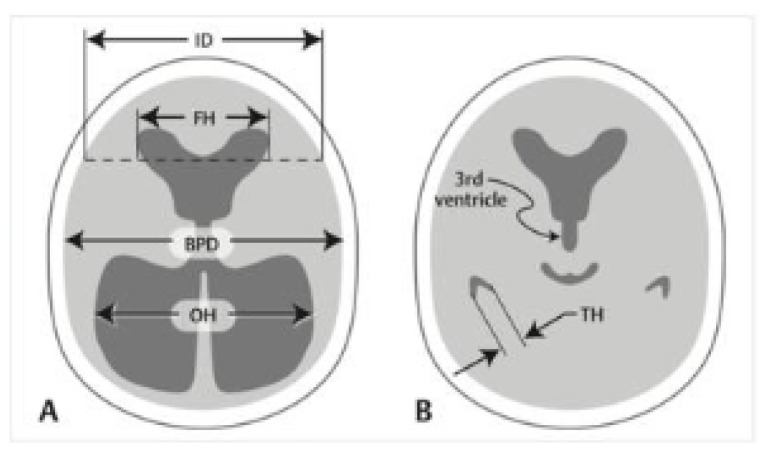
The second method, whereby (B) shows an axial slice for which ventricular measurements for temporal horns of the lateral ventricles are undertaken; which is typically best seen at the level of the petrous apex Note: Adapted from Greenberg (Figure 24.4, pp. 432) (5)

**Figure 6:**
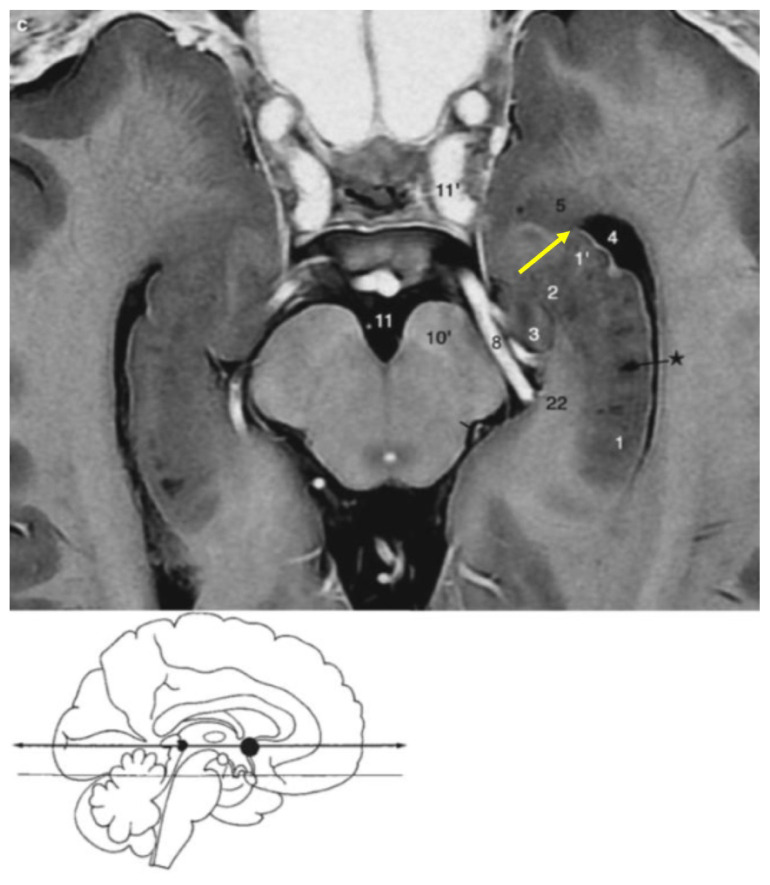
Head of hippocampus, uncal apex, temporal (inferior) horn of the lateral ventricle, amygdala, body of hippocampus and amygdalohippocampal transition area (AHi) Note: Adapted from Duvernoy et al. (Figure 7.26, pp. 212) (4); 1, 2 = head of hippocampus; 3 = uncal apex; 4 = temporal (inferior) horn of the lateral ventricle; 5 = amygdala; black star = body of hippocampus; yellow arrow = amygdalohippocampal transition area (AHi)

**Figure 7 f7-17mjms3201_le:**
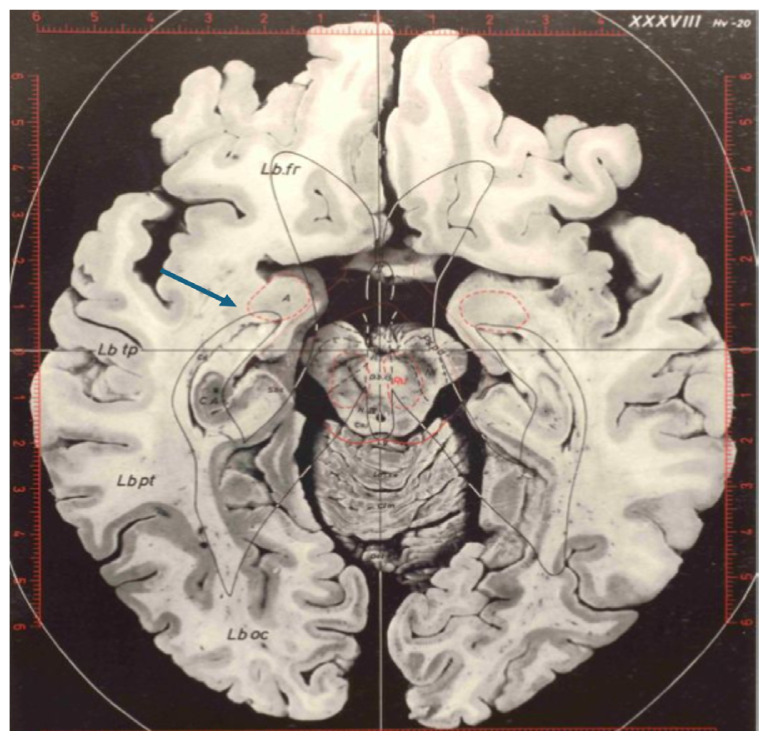
The anterior extent of the temporal horn can be depicted by the amygdalohippocampal transition area (AHi) (blue arrow), which delineates the amygdala (A) from the hippocampus, labelled as CA (cornu Ammonis) Note: Adapted from Schaltenbrand and Wahren (6)

**Figure 8:**
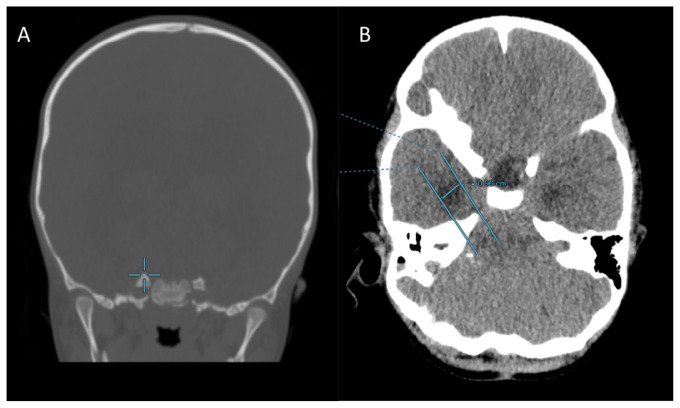
CT images of the similar 3 years old child with grade 4 medulloblastoma as in the case in Figure 4

**Figure 9:**
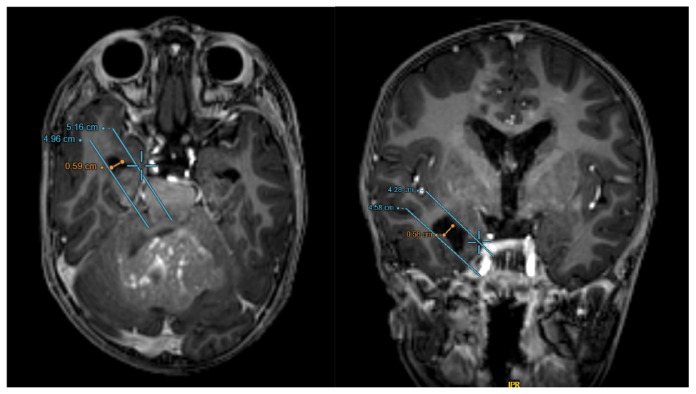
MRI Brain of the similar 3 years old child with grade 4 medulloblastoma as in the case in Figure 4
